# A systematic review and meta-analysis of the pronator quadratus repair following volar plating of distal radius fractures

**DOI:** 10.1186/s13018-020-01942-w

**Published:** 2020-09-16

**Authors:** Chun-Kuan Lu, Wen-Chih Liu, Chung-Chia Chang, Chia-Lung Shih, Yin-Chih Fu, Jesse B. Jupiter

**Affiliations:** 1 Department of Orthopedic Surgery, Kaohsiung Medical University Hospital, Kaohsiung Medical University, Kaohsiung, Taiwan; 2 Ph.D Program in Biomedical Engineering, College of Medicine, Kaohsiung Medical University, Kaohsiung, Taiwan; 3 Department of Orthopedic Surgery, Kaohsiung Municipal Siaogang Hospital, Kaohsiung Medical University, Kaohsiung, Taiwan; 4 School of Medicine, College of Medicine, Kaohsiung Medical University, Kaohsiung, Taiwan; 5 Hand and Upper Extremity Service, Department of Orthopedic Surgery, Massachusetts General Hospital, Harvard Medical School, Boston, MA USA

**Keywords:** Pronator quadratus, Distal radius fracture, Volar plate, Pronation

## Abstract

**Background:**

Distal radius fracture (DRF) is the most common upper extremity fracture that requires surgery. Operative treatment with a volar locking plate has proved to be the treatment of choice for unstable fractures. However, no consensus has been reached about the benefits of pronator quadratus (PQ) repair after volar plate fixation of DRF in terms of patient-reported outcome measures, pronation strength, and wrist mobility.

**Methods:**

We searched the PubMed, Embase, Cochrane Central, and China National Knowledge Infrastructure (CNKI) databases up to March 13, 2020, and included randomized-controlled, non-randomized controlled, or case-control cohort studies that compared cases with and without PQ repair after volar plate fixation of DRF. We used a random-effects model to pool effect sizes, which were expressed as standardized mean differences (SMDs) and 95% confidence intervals. The primary outcomes included Disabilities of the Arm, Shoulder, and Hand scores and pronation strength. The secondary outcomes included the SMDs in pain scale score, wrist mobility, and grip strength. The outcomes measured were assessed for publication bias by using a funnel plot and the Egger regression test.

**Results:**

Five randomized controlled studies and six retrospective case-control studies were included in the meta-analysis. We found no significant difference in primary and secondary outcomes at a minimum of 6-month follow-up. In a subgroup analysis, the pronation strength in the PQ repair group for AO type B DRFs (SMD = − 0.94; 95% CI, − 1.54 to − 0.34; *p* < 0.01) favored PQ repair, whereas that in the PQ repair group for non-AO type B DRFs (SMD = 0.39; 95% CI, 0.07–0.70; *p* = 0.02) favored no PQ repair.

**Discussion:**

We found no functional benefit of PQ repair after volar plate fixation of DRF on the basis of the present evidence. However, PQ muscle repair showed different effects on pronation strength in different groups of DRFs. Future studies are needed to confirm the relationship between PQ repair and pronation strength among different patterns of DRF.

**Registration:**

This study was registered in the PROSPERO registry under registration ID No. CRD42020188343.

**Level of evidence:**

Therapeutic III

## Introduction

Distal radius fracture (DRF) is the most common upper extremity fracture that requires surgery. Operative treatment with a volar locking plate has proved to be the treatment of choice for unstable fractures [1]. The pronator quadratus (PQ) muscle resides in the fracture zone and implant placement site. Several published studies have addressed methods of preservation or repair of the PQ muscle [2–4]. A previous study surveyed all active members of the American Society for Surgery of the Hand in the USA, and 83% (608/753) responded that they attempted to repair the PQ muscle [5]. In addition, one previous biomechanical study that included healthy volunteers reported that subjects with decreased pronation torque strength had temporary pronator quadratus paralysis [6]. However, the necessity for repair of the PQ for optimal functional outcome remains controversial.

Two studies, a retrospective case-control study [7] and a prospective randomized controlled study [8], both published in 2013, compared PQ repair with no PQ repair after distal radius plating surgery. They found no significant differences in Disabilities of the Arm, Shoulder, and Hand (DASH) and pain scale scores 1 year postoperatively. An additional prospective randomized controlled study revealed that PQ repair might reduce pain in 3 months postoperatively [9]. The most recently published randomized controlled trial concluded that PQ repair showed no significant improvement in clinical outcome 1 year after surgery [10].

To compile the best available evidence, we performed a systemic review and meta-analysis of prospective randomized controlled and retrospective case-control studies to determine whether PQ repair after distal radius plating surgery is associated with patient-reported outcome measures, pain scale score, wrist mobility, and grip and pronation strengths.

## Methods

### Search strategy and inclusion criteria

We conducted this study in accordance with the Preferred Reporting Items for Systematic Reviews and Meta-Analyses guidelines [11]. We performed an electronic search in the PubMed, Embase, Cochrane Central, and China National Knowledge Infrastructure (CNKI) databases up to March 13, 2020, using search strategies (Additional file 1 Appendix 1). The bibliographies of the included trials and related review articles were manually reviewed for relevant references. Two independent reviewers (CCC and CKL) evaluated the studies by screening the titles and abstracts, followed by a detailed examination of the full texts of the eligible articles. Any inconsistencies were resolved using a consensual approach. If a disagreement could not be resolved, we consulted a third reviewer (WCL) for the final decision.

**Fig. 1:**
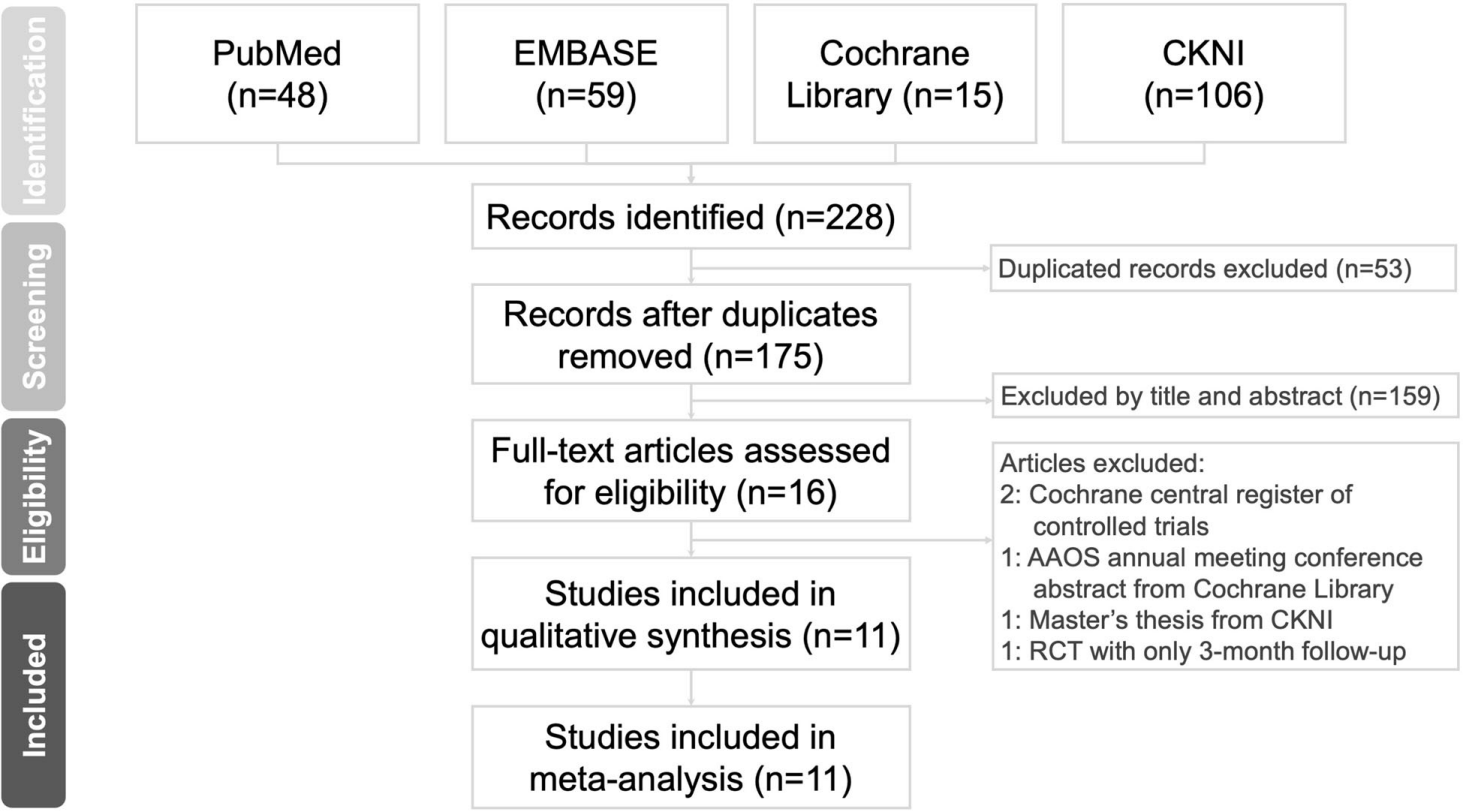
PRISMA flowchart. Preferred Reporting Items for Systematic Reviews and Meta-analyses flow diagram for searching and identifying the studies for inclusion in the analysis

Regarding the types of studies included, we enrolled randomized controlled trials (RCTs) and comparative experimental trials. We included clinical trials that met the following criteria: (1) included a target population that was comprised of patients with DRFs treated with volar plate fixation; (2) comprised of two treatment arms after volar plate fixation, with and without PQ repair; and (3) measured the clinical outcome at least 6 months postoperatively. We excluded the following types of studies: (1) reviews, conference abstracts, or presentations, and (2) overlapping publications. We summarized the selection process in accordance with the PRISMA flowchart (Fig. 1).

### Methodological quality assessment

All 12 studies were critically appraised for the assessment of their methodological qualities by two independent reviewers (WCL and CKL), which was checked by a third reviewer (CLS). We recorded the first author, year, number of fracture patterns based on the AO classification, participant characteristics, model of the volar plate, and detailed technique of the PQ repair. The methodological qualities of the enrolled studies were evaluated by two reviewers independently using the Jadad scale for RCTs [12] and the Newcastle-Ottawa Scale (NOS) for comparative experimental trials [13]. The Jadad scale evaluates the methodology of RCTs in accordance with three aspects as follows: randomization (2 points), blinding (2 points), and an account of all patients (1 point) [12]. The five RCTs included in our study ranged had Jadad scale scores ranging from 3 to 5, with a maximum possible score of 5. Higher scores indicate better methodological quality. In evaluating case-control studies, the NOS contains nine items in three categories as follows: participant selection (four items), comparability (one item), and exposure (three items). A study can be scored a maximum of 1 point for items in the selection and exposure domains and a maximum of 2 points for the comparability domain [14]. The five case-control studies included all had a NOS score of 8, with a maximum possible score of 9. Higher scores indicate better methodological quality. Between-reviewer discrepancies were resolved through discussions under the supervision of the corresponding author. We tried to contact the primary authors of all the included studies; however, only the authors of three studies responded and provided complete original data sheets [7, 8, 10].

### Meta-analysis methodology

A meta-analysis was performed using the Comprehensive Meta-Analysis (CMA) version 3 software (Biostat, Englewood, NJ, USA), thereby combining the relevant effects of interest from our identified studies.

All the outcomes were assessed within two groups after volar plate fixation of DRF as follows: PQ repair was performed in the study group but not in the control group in a minimum of 6 months after distal radius plating surgery. The standardized mean differences (SMDs) in DASH score and pronation strength between the two groups were the primary outcomes. The SMDs in wrist mobility (extension, flexion, supination, pronation, radial deviation, and ulnar deviation) and grip strength and visual analog scale score for pain between the groups were the secondary outcomes. A negative SMD value indicated that PQ repair was a favorable treatment option. Studies that did not report standard deviations (SDs) were excluded from the pooling. We also recorded any flexor complications mentioned in each included study. When the 95% confidence interval (CI) of the summary mean did not overlap, we considered it statistically significant. Between-trial heterogeneity was determined using *I*^2^ and chi-square tests [15]. A fixed-effects (inverse variance) model was used when the effects were assumed to be homogenous (*p* > 0.01). Statistical heterogeneity is implied when the *p* value was < 0.01; thus, a random-effects model was used in those circumstances. Articles that reported outcome measures were assessed for publication bias using a funnel plot [16] and the Egger regression test [17].

## Results

### Literature search and study characteristics

We retrieved 175 non-duplicate citations and reviewed their titles and abstracts, and included 15 articles for meticulous evaluation after eliminating references that did not meet the inclusion criteria (Fig. 1). We excluded two studies from the Cochrane Central Register of Controlled Trials database, one master’s thesis, which is a review article from the CNKI database, and one RCT that compared PQ repair with no PQ repair with only 3 months of follow-up. Therefore, the meta-analysis included five RCTs [8, 10, 18–20] and six retrospective case-control studies [7, 21–25].

A total of 732 patients were included along with a breakdown of the numbers of patients, mean ages, and sex ratios in comparison groups, except for two studies. Patient sex data and mean patient age were well recorded in all the studies. The mean ages of the patients ranged from 48.7 to 64.0 years in the group with PQ repair and from 47.1 to 63.6 years in the group without PQ repair. The female-to-male ratio in each study was well proportioned between the two study groups. The mean patient age and sex ratio were also comparable in all the studies. The details of each study are summarized in Table 1.

**Table 1:** Summary of the retrieved studies that compared between with and without pronator quadratus repair after distal radius fracture volar plating surgery

Study (author, year)	Study design	Sex (M/F)	Fracture stage (A/B/C)	Mean age, years	Blinding	Randomization	Outcome measures	Follow-up timing	Country	Quality assessment
Si, 2012	RCS	R: 15 (3/12) NR: 15 (5/10)	R: 0/15/0 NR: 0/15/0	R: 57.6 (35–70) NR: 56.3 (48–70)	NA	NA	DASH and ROM	3 days, 1 month, and 6 months	China	8^#^
Hershman, 2013	RCS	R: 62 (29/33) NR: 50 (21/29)	R: 13/10/29 NR: 17/12/21	R: 53.8 (4) NR: 51.6 (4.5)	NA	R: hand fellow; NR: trauma fellow	DASH, VAS score, grip strength, and ROM	6 weeks, 3 months, 6 months, and 12 months	USA	8^#^
Tosti, 2013	RCT	R: 33 (9/24) NR: 24 (4/20)	R: 8/1/24 NR: 2/1/21	R: 51 (18.9) NR: 60 (13.7)	Double	Year of birth	DASH, VAS, ROM, and grip strength	2 weeks, 6 weeks, 3 months, and 12 months	USA	3*
Qian 2017	RCS	R: 43 (NA) NR: 40 (NA)	R: 0/21/22 NR: 0/20/20	R & NR: 52.2 (23–72)	NA	NA	DASH, ROM, and pronation/supination power	13 months (11–19 months)	China	8^#^
Hohendorff, 2018	RCT	R: 20 (4/16) NR: 16 (6/10)	R: 14/0/6 NR: 8/1/7	R: 64 (18–77) NR: 54 (18–80)	NA	Blinded envelope	DASH, VAS, ROM, grip strength, and pronation power	12 months	Germany	4*
Wang, 2018	RCS	R: 25 (4/21) NR: 20 (4/16)	R: 0/12/13 NR: 0/8/12	R: 53.6 NR: 57.8	NA	NA	DASH, VAS, and ROM	6 weeks and 6 months	China	8^#^
Zhang, 2018	RCT	R: 45 (29/16) NR: 45 (30/15)	R: 5/22/18 NR: 6/19/16	R: 55.7 (3.4) NR: 54.1 (2.4)	Double	Admission sequence	VAS and ROM	1 day, 3 days, 1 month, and 6 months	China	2*
Cang, 2019	RCS	R: 37 (NA) NR: 23 (NA)	R: 0/18/19 NR: 0/12/11	R & NR: 40.9 (5.0)	NA	NA	ROM, DASH and pronation/supination power	10.5 months (6–15 months)	China	8^#^
Chao, 2019	RCT	R: 42 (13/29) NR: 42 (15/27)	R: 9/25/8 NR: 7/24/11	R: 48.7 (7.7) NR: 47.1 (8.5)	Single	Undescribed method	DASH, VAS, ROM, and grip strength	2 weeks, 6 weeks, 3 months, and final (11–15 months)	China	2*
Pathak, 2019	RCS	R: 29 (11/18) NR: 34 (13/21)	R & NR: A2–C2	R: 54.9 (24–77) N: 48.6 (22–72)	NA	NA	DASH, VAS, ROM, and grip strength	1 month, 3 months, 6 months, and final (R: 35.2; NR: 38.6)	India	8^#^
Sonntag, 2019	RCT	R: 36 (5/31) NR: 36 (10/26)	R: 22/0/12 NR: 19/1/13	R: 62.0 (10.8) NR: 63.6 (15.6)	Double	Randomized allocation sequence	DASH, PRWE, ROM, pronation, and grip strength	2 weeks, 5 weeks, 3 months, 6 months, and 12 months	Denmark	5*

*RCS* retrospective case-control study, *RCT* randomized controlled trial, *R* repair, *NR* non-repair, *NA* not applicable, *DASH* Disabilities of the Arm, Shoulder, and Hand, *ROM* range of motion, *VAS* visual analog scale, *MWS* Mayo wrist score, *PRWE* patient-rated wrist evaluation

^#^The study was assessed using the Newcastle–Ottawa scale score

*The study was assessed using the Jadad scale score

For pronation strength measurement, two studies [10, 18] used a baseline hydraulic wrist dynamometer (Fabrication Enterprises), and another two studies [25] used a hand dynamometer (Qianli, China). For grip strength measurement, three studies [8, 10, 18] used a dynamometer (Jamar; Therapeutic Equipment, Clifton, NJ) and two studies [7, 19] did not mention the hand dynamometer model used. One study [8] provided grip strength data in comparison with those of the uninjured side instead of the actual measured values.

Most studies presented the number of fractures in each AO Foundation/Orthopaedic Trauma Association (AO/OTA) fracture classification [26], except one study [23] that did not report the included fracture types. Two studies analyzed the outcomes of patients with DRF AO/OTA types B and C [22, 25] with or without PQ repair instead of the pooled outcomes. We divided these two studies into two sub-studies for the statistical analysis.

### Surgical procedure and postoperative management

All the internal fixation surgeries were performed using the modified volar approach of Henry. Among the included studies, different types of volar plate, methods of PQ repair, and postoperative management strategies were used. We summarized them in Table 2.

**Table 2:** Summary of the surgery and postoperative management details of the retrieved studies

Study (author, year)	Plate	PQ repair method	Postoperative management
Si, 2012	Unknown VLP	Direct interrupted (absorbable 2-0)	Physical therapy in 2 days
Hershman, 2013	Stryker and Synthes VLP	NA	Splint for 2 weeks, full weight bearing in 6 weeks
Tosti, 2013	Medartis (VA) and Synthes (VA) VLP	Figure-of-eight (Vicryl 2-0)	Immobilization for 2 weeks and then start of physical therapy
Qian 2017	Synthes VLP	Figure-of-eight (Vicryl 3-0)	NA
Hohendorff, 2018	Stryker (VA) VLP	PQ to BR (PDS 4-0)	Splint for 2 weeks and unlimited mobility in 4 weeks
Wang, 2018	NA	Figure-of-eight (absorbable 4-0)	Physical therapy in 2 days
Zhang, 2018	Unknown VLP	Direct interrupted (absorbable 2-0)	Physical therapy in 2 days
Cang, 2019	Synthes VLP	Figure-of-eight (absorbable 3-0)	NA
Chao, 2019	NA	Direct interrupted (absorbable 3-0)	Physical therapy in 1 weeks
Pathak, 2019	NA	Direct interrupted (Vicryl 3-0)	Immobilization for 1–2 weeks and then start of physical therapy
Sonntag, 2019	Stryker (VA), Synthes (VA)	Continuous with a minimum of four sutures (Vicryl 3-0)	Splint for 2 weeks and gradual weight bearing

*PQ* pronator quadratus, *NA* not applicable, *VLP* volar locking plate, *VA* variable angle, *PDS* polydioxanone

### Meta-analysis of the outcomes

**Fig. 2:**
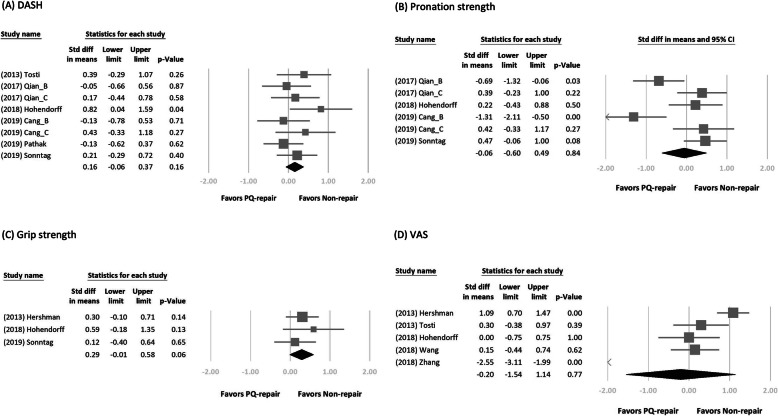
Forest plots of the standardized mean differences in functional outcome, wrist strength, and pain score. Forest plots of the standardized mean differences in **a** Disabilities of the Arm, Shoulder, and Hand score, **b** pronation strength, **c** grip strength, and **d** visual analog scale score for pain between the two treatment arms

**Fig. 3:**
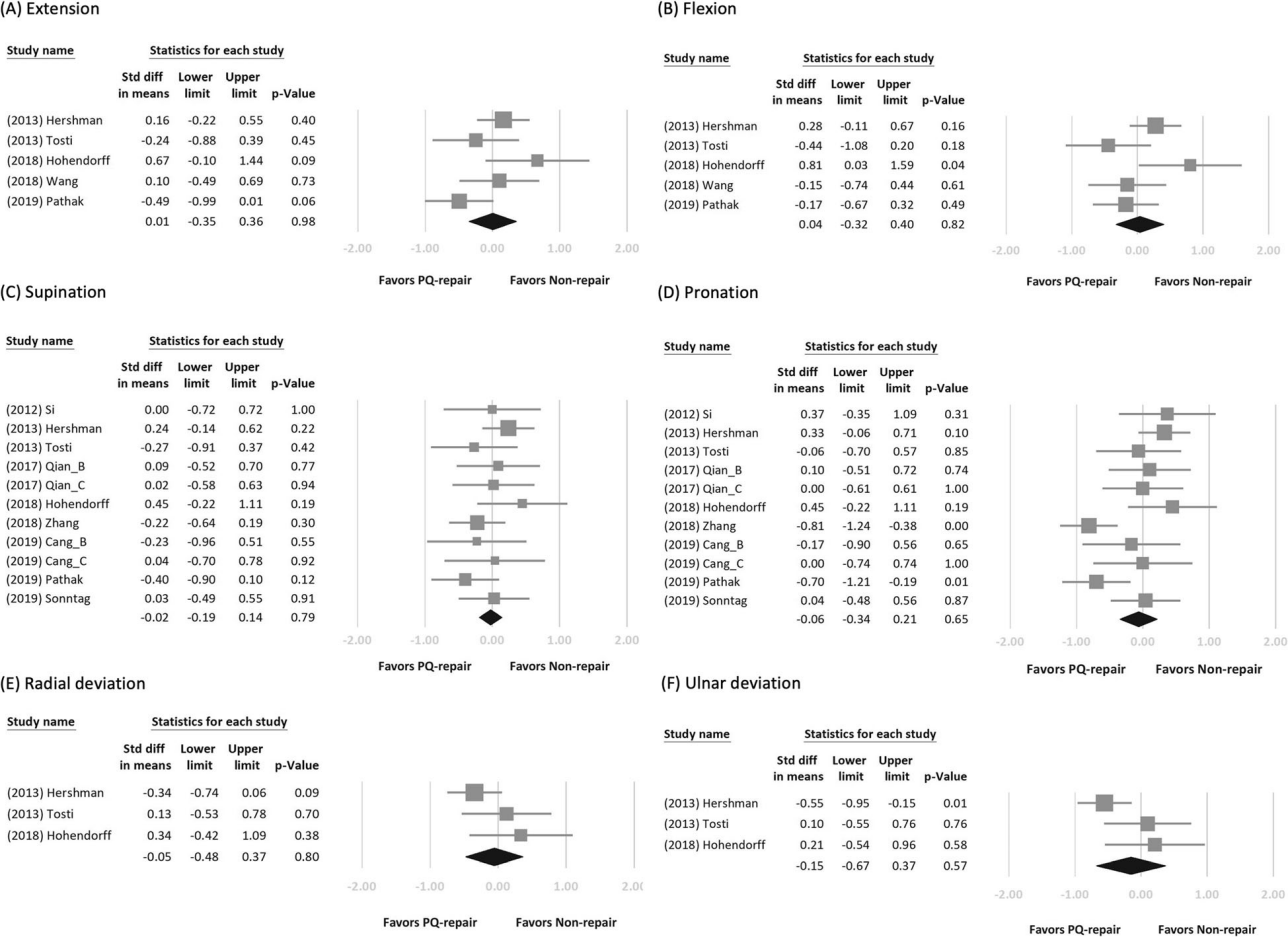
Forest plots of the standardized mean differences in wrist mobility. Forest plots of the standardized mean differences in wrist mobility between the two treatment arms: **a** extension, **b** flexion, **c** supination, **d** pronation, **e** radial deviation, and **f** ulnar deviation

**Fig. 4:**
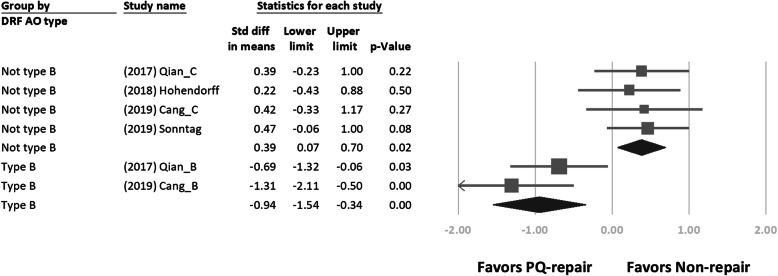
Forest plots of the standardized mean differences in pronation strength. Forest plots of the standardized mean differences in pronation strength between the two treatment arms for AO type B distal radius fractures

We retrieved the long-term follow-up outcome from each study. Figures 2 and 3 show the forest plot of the different clinical outcomes between the two groups. We demonstrated the pooled mean of the outcomes in Table 3. No significant difference in each outcome was found between the two treatment arms. In a subgroup analysis of DRFs of AO type B or non-type B, we found a significant difference in pronation strength between the two treatment arms (SMD = − 0.94; 95% CI, − 1.54 to − 0.34; *p* < 0.01, favoring PQ repair in the type B group vs SMD = 0.39; 95% CI, 0.07–0.70; *p* = 0.02, favoring PQ repair in the non-type B group; Fig. 4).

**Table 3:** Summary of the outcomes by meta-analysis

	With PQ repair			Without PQ repair		
	Pooled estimate	95% CI	*n*	Pooled estimate	95% CI	*n*
DASH	24.73	16.22–33.25	8*	22.15	9.86–34.44	8*
Pronation strength (kg cm)	50.70	44.32–67.08	6*	57.39	40.32–74.45	6*
Grip strength (kg)	32.63	19.86–45.39	3	32.86	19.77–45.94	3
VAS	1.39	0.89–1.89	5	1.61	0.40–2.81	5
Extension (degrees)	62.84	45.09–80.59	6	63.94	52.31–75.57	6
Flexion (degrees)	62.18	48.99–75.36	6	63.23	53.05–73.41	6
Supination (degrees)	68.12	53.82–82.42	11*	68.66	53.63–83.69	11*
Pronation (degrees)	62.86	52.24–73.24	11*	63.00	52.36–73.64	11*
Radial deviation (degrees)	19.07	16.52–21.62	3	20.67	18.95–22.39	3
Ulnar deviation (degrees)	31.42	25.60–37.25	3	33.98	31.45–36.51	3

*PQ* pronator quadratus, *SMD* standardized mean difference, *CI* confidence interval, *DASH* Disabilities of the Arm, Shoulder, and Hand, *VAS* visual analog scale for pain

*Presented outcomes separately for AO type B and C fractures, which we counted as two studies

The Egger test revealed no significant publication bias regarding most clinical outcomes except radial deviation range of motion (*t* = 39.2, *df* = 1, *p* = 0.02; Table 4).

**Table 4:** Results of the Egger test for each outcome

	*t*	*df*	*p* value
DASH	2.08	6*	0.082
VAS	0.15	3	0.891
Extension	0.27	3	0.802
Flexion	0.09	3	0.931
Supination	0.05	9*	0.958
Pronation	0.70	9*	0.500
Radial deviation	39.2	1	0.016
Ulnar deviation	9.29	1	0.068
Grip strength	0.64	1	0.637
Pronation strength	1.36	4*	0.244

*DASH* Disability of Disabilities of the Arm, Shoulder, and Hand score, *VAS* visual analog scale

*Presented outcomes separately for AO type B and C fractures, which we counted as two studies

## Discussion

With the popularity of the volar locking plate in the management of unstable DRFs, demonstrable repair of the PQ muscle postoperatively has become an issue. The PQ consists of a superficial head, which acts on forearm pronation, and a deep head, which is a dynamic stabilizer of the distal radioulnar joint [27]. As noted, a clinical study in healthy volunteers demonstrated that pronation strength decreased by 21% after the PQ muscle was anesthetized [6]. However, most clinical studies that compared cases with and without PQ repair after distal radius volar plating surgery did not show this significant difference.

Regarding pronation strength, two studies showed that the pronation strength in the group without PQ repair decreased significantly only in the patients with AO/OTA type B fractures but not in those with AO/OTA type C fractures [22, 25]. One study included patients with AO/OTA type A2, A3, and C1 fractures [18], while another study included patients with AO/OTA type A2, A3, C1–3 fractures [10]. Having better repair quality with preoperatively intact and nicely prepared PQ flaps seems logical. Although the durability of PQ repair has been supported [5], ensuring good-quality repair, especially in comminuted DRFs with frayed PQ muscles, is sometimes difficult. Two RCTs routinely checked the length or retraction of the PQ in ultrasonography examinations [10, 18]. In our included studies, patients in the group without PQ repair who had more metaphyseal displaced and complex fracture patterns had better pronation strengths (Fig. 4). However, a retrospective study showed that the completeness of PQ repair did not influence wrist mobility and grip strength [28]. More evidence is needed to confirm the relationship between the quality of PQ repair and pronation strength among different patterns of DRF.

The reliability and validity of the DASH questionnaire in the German, Chinese, and Danish versions have been confirmed [29–31]. Fracture classification might influence clinical outcomes. We found that most studies included patients with similar distributions of AO/OTA types A, B, and C. As a result, this factor was controlled within each group. The other factor is postoperative treatment. Each study had a different postoperative treatment. An RCT found that patients starting wrist mobilization 2 or 6 weeks after volar plate fixation of the distal radius did not influence the patient-reported outcome measures, grip strength, and wrist mobility at 6 and 12 months [32]. Although each study used varied postoperative treatments, we could assume that it did not influence the postoperative outcome in a minimum of 6 months for quantitative calculation.

This systematic review is limited by the sample size and quality of the available studies. To compensate for limiting the research articles in English, we conducted an extensive search strategy in most available databases, including CNKI, to ensure that all potentially relevant papers were identified and reviewed. The measurement tools for grip and pronation strength varied greatly in the included studies, which could influence the data quantification. Finally, three authors provided raw data, making up for the missing items in the included studies [7, 8, 10].

One potential benefit of PQ repair is that it enables the separation of the volar plate from the flexor tendon, which might prevent complications such as flexor tendon irritation or rupture. A systematic review revealed that the median interval between surgery and flexor tendon rupture was 9 months (interquartile range, 6–26 months) [33]. A cohort study included 451 patients with a mean follow-up of 3.2 years. The flexor tendon rupture rate was only 1.1% [34]. In the studies included in our analysis, no flexor tendon rupture was found in each group. The discrepancy in the literature may be attributed to the difference in follow-up duration. We could not conclude whether PQ repair is a protective factor against flexor complications. Future studies with longer follow-up periods are needed to compare between with and without PQ repair.

## Conclusion

This systematic review and meta-analysis found no significant differences between the groups with and without PQ repair in terms of DASH score, pronation strength, pain score, wrist mobility, and grip strength at a minimum of 6-month follow-up after volar plating surgery for DRF. However, PQ muscle repair showed different effects on pronation strength among the different DRF groups. Future studies are needed to confirm the relationship between PQ repair and pronation strength among the different DRF patterns.

## Supplementary information

**Additional file 1:.** Search query

## Data Availability

The datasets used/or analyzed during the current study are available from the corresponding author on reasonable request.

## Abbreviations

AO/OTA: AO Foundation/Orthopaedic Trauma Association
CI: Confidence interval
DASH: Disability of Disabilities of the Arm, Shoulder, and Hand score
DRF: Distal radius fracture
NOS: Newcastle-Ottawa Scale
PQ: Pronator quadratus
RCT: Randomized controlled trial
SMD: Standardized means difference
VAS: Visual analog scale for pain
