# Supplementary material for: A systematic review and meta-analysis of the pronator quadratus repair following volar plating of distal radius fractures
Source: J Orthop Surg Res. 2020 Sep 16;15:419. doi: 10.1186/s13018-020-01942-w (PMC7493143; doi:10.1186/s13018-020-01942-w)
Supplement: Supplementary file 1 — Additional file 1:. Search query [file 13018_2020_1942_MOESM1_ESM.docx]

| Appendix 1. Search query | | | | | |
| --- | --- | --- | --- | --- | --- |
| Date | Database | Clarification | Search | Query | Items found |
| March 13^th^, 2020 | PubMed | [tiab] = words in title or abstract | #1 | distal radi* | 9,553 |
|  |  |  | #2 | volar plat*[Title/Abstract] | 906 |
|  |  |  | #3 | pronator quadratus[Title/Abstract] | 323 |
|  |  |  | #4 | #1 AND #2 AND #3 | **48** |
|  | Embase | /exp = EMtree keyword with explosion  :ab,ti = words in title or abstrac | #1 | **distal** AND (**'radius'**/exp OR **radius**) AND (**'fracture'**/exp OR **fracture**) | 9,215 |
|  |  |  | #2 | **volar**:ab,ti AND **plat***:ab,ti | 1,658 |
|  |  |  | #3 | **pronator**:ab,ti AND **quadratus**:ab,ti | 371 |
|  |  |  | #4 | #1 AND #2 AND #3 | **59** |
|  | Cochrane Library | :ti,ab,kw = words in title, abstract or keyword | #1 | distal radi* | 3,980 |
|  |  |  | #2 | (volar plat*):ti,ab,kw | 253 |
|  |  |  | #3 | (pronator quadratus):ti,ab,kw | 21 |
|  |  |  | #4 | #1 and #2 and #3 | **15** |
|  | CNKI | 篇关摘= title, keyword and abstract | #1 | 桡骨远端骨折 and 旋前方肌: 篇关摘 | **106** |
